# Gambling advertising regulation in Ghana: what do we know and where to next?

**DOI:** 10.1057/s41271-025-00547-z

**Published:** 2025-01-17

**Authors:** Emmanuel Badu, Gemma Crawford, Jonathan Hallett, Justine E. Leavy

**Affiliations:** 1 Collaboration for Evidence Research and Impact in Public Health, School of Population Health, Curtin University, Perth, WA Australia; 2 School of Population Health, Curtin University, Perth, WA Australia

**Keywords:** Gambling, Advertising, Ghana, Betting, Sub-Saharan Africa, Commercial determinants

## Abstract

In Ghana and many other sub-Saharan African countries gambling advertising has become pervasive due to weak regulations that allow gambling operators to zealously promote their products as a risk-free way to make money. In this commentary, we provide a public health perspective based on document analysis of the Ghana Gaming Commission’s guidelines on advertisements, and recommendations for strengthening Ghana’s gambling regulatory environment. With the industry intensifying its focus on new markets to grow and sustain profits, and new global players entering Ghana’s market, the competition for market share will most likely intensify with an associated and aggressive increase in gambling advertising. Accordingly, a strengthened gambling advertising regulation underpinned by public health principles is required to restrict advertising across all forms of media and regulate advertising content. The next step for public health action should focus on advocating for new gambling advertising regulations and a review of the Gaming (gambling) Act.

## Key messages

- The Ghana Gaming Commission’s advertising guidelines privileges individual responsibility and industry self-regulation; they are weak and outdated.
- To prevent and minimize gambling harms, a strengthened gambling advertising regulation is urgently required to restrict advertising on all media platforms and to regulate advertising content.
- Public health action must focus on advocating for gambling regulatory reforms.

## Introduction

Globally, commercial gambling advertising has increased in intensity, interactivity, and complexity in the last decade [1]. In sub-Saharan Africa (SSA), the increase in commercial gambling advertising is largely a result of legislation not keeping pace with technological advancements such as the transition from traditional media to online advertising, and industry innovation including the rapid evolution of diverse online gambling products [2]. In addition, Governments in the region have prioritized revenue generation from commercial gambling over their constitutional and international law obligations which are to protect the health of their citizens [3]. Consequently, powerful commercial vested interests have deployed their substantial economic power to influence policy decisions [4] over the public interest in gambling regulations [2, 3]. This has resulted in a gambling industry regulatory environment that is weak and outdated and offers the public little protection from the harmful effects of the liberalized gambling market [2]. The prevailing governments’ inertia to effectively regulate the gambling industry [5, 6] and its activities in SSA drives gambling harms, including adverse financial impacts, harm to relationships, emotional and psychological distress and impacts to physical health [7].

In Ghana, increased gambling advertising can be linked to the enactment of the Gaming Act 2006 (Act 721), which legalizes gambling and permits all forms of gambling and gambling product advertising. Prior to the Gaming Act 2006 (Act 721), gambling advertising was restricted. The introduction of the Act legitimized gambling as part of the entertainment sector and allowed operators to advertise while placing little limitations on advertising. Consequently, gambling advertisements are ubiquitous [8]. Ghana’s gambling advertising regulation has lagged the rapid evolution of the industry, creating an industry-friendly environment that has enabled gambling operators to vigorously promote gambling as a risk-free way to make legal money [2, 3]. The evidence on the impact of gambling advertising in Ghana is lacking, however research on the public health impact of gambling advertising in other countries [9], suggests that advertising may shape permissive sociocultural environments and influence norms and attitudes towards gambling. Accordingly, this commentary aims to provide a contemporary perspective on Ghana Gaming Commission’s guidelines on advertisement. It applies a comprehensive public health approach (articulated below) [10] to review Ghana’s guidelines on gambling advertisements and offers recommendations for strengthening the gambling regulatory environment.

## Applying a public health approach to gambling advertising regulation

A comprehensive public health approach to gambling has been championed by public health scholars and advocates as a useful guide for preventing and minimizing gambling harm [10]. This approach recognizes the complex interactions between commercial, political, economic, social and behavioral determinants influencing gambling harms and prioritizes policies that protect the health and safety of populations from the harmful effects of the gambling industry [10]. It acknowledges the limitations of individual responsibility approach, ensuring that government creates policies that promote public’s health and wellbeing. When applied to gambling advertising regulations, a comprehensive public health approach would require governments to proactively regulate the gambling industry including the introduction of a comprehensive ban on all forms of advertising of gambling products to protect people from gambling related harm [10].

In practice, regulatory responses to gambling advertising may include: (1) restricting exposure to advertisements by prohibiting gambling advertising; (2) applying zoning measures to minimize exposure to minors or vulnerable people such as restricting advertising during certain times of the day; and (3) imposing standards to guide advertising content to reduce harmful exposure [11]. Existing research suggests governments’ preference for options two and three and similar policies that focus on individual vulnerabilities which engender gambling behavior and targeted interventions for those individuals [12]. We would argue that individualized behavioral policy solutions absolve the industry and governments of any responsibility and have been shown to be less effective in preventing and protecting people from gambling and gambling related harm [6, 10, 13].

## The Ghanaian context

Over the last decade, the rapid expansion of gambling in Ghana has offered the gambling industry significant resources to develop and implement sophisticated advertising campaigns. In 2024, revenue from online gambling alone is projected to rise from about USD 27million (USD 15million from sports betting) in 2019 to about USD 57million (USD 30million from Sports betting), making Ghana the fifth biggest online gambling market in Africa [14]. With large global brands entering the market in 2022, the competition for market share will most likely intensify with an associated increase in gambling advertising.

In Ghana, accessible information on gambling industry’s expenditure on gambling advertising is lacking. However, the Ghana Sports Betting Association, the umbrella body for all licensed sports betting operators, claims expenditure of approximately GHS200 million (equivalent to about 17million USD) annually on advertising [15]. While official requests by the authors of this article to the Ghana Gaming Commission for information about market size and share by gambling operators, revenues and advertising expenditure have not been forthcoming, a recent media report indicated the projected tax revenue from the entire gambling industry for the 2024 financial year as GH¢1.7 billion (equivalent to US $141million) [16].

Ghana’s gambling advertising landscape is not dissimilar from that of other countries. Despite the evidence of harm associated with gambling to individuals, families and communities [17, 18], the gambling industry continues to market its products as enjoyable leisure activities with little or no reference to the harmful consequences of gambling or the actual odds of winning a bet [19–21]. Advertising content and narratives are country specific. However, the available evidence suggests that gambling operators may employ similar themes to influence gambling behavior [19], for example, wealth, cultural celebrations, luxury, fun, thrills of winning and enjoyment [21], albeit adapted to local sociocultural contexts [22, 23]. Such themes create a distorted perception of gambling and social identity cues for the public, particularly for young people and other vulnerable groups [21]. In addition to their demonstrated appeal, gambling advertisements create an illusory sense of consumer control [24]. In this context, advertisements are complex prescriptions of imaginary lifestyles filled with excitement and the financial resources to sustain a dream life [21].

Gambling advertising occurs across different media platforms, including traditional media, digital and social media platforms [1]. Online advertising, including social media and other digital platforms, remains largely free from effective regulation [1]. This means gambling operators can have unfettered access to their consumers and the general population [25], including children and young people [26]. While sanitizing or removing advertising in traditional media environments such as television and radio may play an essential role in preventing gambling initiation, the shift to, and extent of advertising online means that young people and other vulnerable groups remain highly exposed without reforms for strong regulation [1].

## Gambling advertising regulation in Ghana

The Gaming Commission of Ghana (the Commission), which regulates gambling in Ghana, developed guidelines for gambling advertising in Ghana in 2020 [27] (see Box 1).

Box 1. Summary of the Ghana Gaming Commission’s gambling advertisement guidelines The guidelines aim to ensure that all advertisements by operators are “devoid of deception and minimize exposure to minors” [27]. The guidelines state the Commission must approve all gambling advertisements, must acknowledge the Commission as the regulator of gambling; shall not use celebrities or create the impression of assured wins, and shall not depict or encourage excessive gambling; and must contain responsible gambling warnings such as ‘gambling responsibly, gambling is addictive, gambling is only for those 18 years and above’ [27]. The guidelines emphasize radio and television advertising, noting advertising bans at prime time (prime time not defined in the guidelines). There is little mention of digital, online, or social media advertising in the guidelines. Characters in advertisements cannot be depicted as wealthy or imply that characters acquired their wealth through gambling or that gambling is a source of regular income for characters. Regarding sponsorship, guidelines only apply to the advertisement or publicity of events where organizers cannot distribute marketing materials to persons under 18 and must submit any sponsorship advertising material for a program or sporting event to the Commission for approval. This does not include sponsorship agreements with other organizations, such as sports clubs. Further, the guidelines prohibit advertising targeted at persons less than 18 years of age. There is a specific requirement that operators not appeal directly or indirectly to underage persons in the use of children’s media, children’s songs, or cartoon characters. The guidelines prohibit the placement of outdoor advertising within 200 m of schools and at functions where underage persons are likely to attend [27].

## Gaps in Ghana’s advertising regulation

Applying a public health approach, we identified three main gaps in the current advertising regulatory framework: (1) the guidelines prioritize individual behavioral solutions in common with the dominant responsible gambling approach (2) the guidelines do not address online gambling advertising (3) the guidelines lack a monitoring and enforcement framework. These gaps are further discussed below.

As indicated in box 1, gambling advertising regulation in Ghana privileges individual responsibility and industry self-regulation. This regulatory approach exemplifies the fundamental conflict between liberalizing gambling advertising and the state’s role in protecting its citizens, particularly young people, and other vulnerable groups from harm [11]. As demonstrated across a range of public health issues that relate to harmful commodities such as alcohol, tobacco and ultra-processed food, self-regulation often results in no or ineffective regulation [28] and does not guarantee effective harm minimization [4].

Ghana government’s approach to regulating the gambling industry reflects a broader sentiment regarding the expansion of commercial practices as a solution to a range of economic and health challenges, particularly in the context of revenue generation necessary for debt servicing and social service provisioning [4, 5]. However, the industry’s market justice and profit aspirations directly oppose the social justice public health agenda of preventing and minimizing harm [5, 6]. For instance, a recent study in South Africa found that food companies increased their spending on advertisements and failed to adhere to voluntary pledges and codes to protect children from advertisements [29]. Similar findings from Malawi have demonstrated the sophisticated strategies the gambling industry employs to grow the sports betting market rapidly over a short period [23]. Thus, a strong regulatory framework that incorporates a clear, proactive routine monitoring and enforcement mechanism, is necessary to protect the public from the harmful activities and products of the gambling industry.

As operators shift gambling opportunities from predominantly land-based and traditional activities to online or digital platforms [1] they have concomitantly adopted sophisticated online advertising strategies to attract new customers [25]. For example, around 80% of gambling in Ghana is now reportedly online [16], and findings from a recent survey reported that over 95% of respondents from Ghana primarily placed sports bets via online platforms [30]. However, gambling regulations, including the advertising guidelines, have not kept pace with the rapid technological advances, evidenced by the lack of guidance related to online or social media advertising in Ghana and elsewhere. The lack of online gambling regulations is common across many countries, as many governments have failed to keep pace with online gambling modes entering the market [21, 26]. Across SSA region legislation explicitly addressing online gambling has been identified in 15 out of 41 countries (36.6%) while 18 out of 41 (43.9%) countries have reported legislation for advertising [2]. The online space is rapidly evolving and would require stronger and specific regulations by governments.

The predominance of online gambling advertising also has implications for targeting new and often vulnerable consumers. Young people’s familiarity with the Internet and its wide availability and use increases the risk of exposure to gambling adverts and the likelihood of betting [25, 26]. Operators can deliver more personalized messages using algorithms, which poses a significant threat to young people who gamble [26]. Evidence demonstrates gambling industries deliberately target young people as future loyalists and consumers of their harmful products [21]. Influencer and affiliate marketing on social media are rapidly evolving and present a new opportunity for gambling operators to promote signup promotional offers, bonuses, and free bets to followers of popular social media influencers and content creators [31]. Existing laws in Ghana do provide some safeguards, albeit poorly enforced. For instance, persons aged under 18 years are prohibited from gambling. The Know-Your-Customer (KYC) provisions under the Anti-Money Laundering Act, 2020 (Act 1044) require operators to verify every customer to ensure they meet the age criteria before creating an account. To place a bet online, individuals must own a mobile money account, an electronic wallet on mobile phones for storing and sending money. However, persons under 18 years are not permitted to register for a mobile money account and should not be able to gamble online. Yet, evidence of underage online gambling exists [15], a strong indication of the pervasiveness of gambling advertisements [26]. The Commission’s current guidelines only require operators to screen responsible gambling warnings as part of their online advertising. Guidelines do not specify the content or placement nor provide any restrictions on time and demographic targeting. The changing landscape of gambling in Ghana, from brick-and-mortar outlets to the anonymity and convenience of online forms, poses the most significant threat yet to young people’s gambling initiation and subsequent risks for health and social harms.

## Policy implications

Gambling advertising in all its forms has become inescapable in Ghana, raising important questions about industry practices and government oversight at the national level. The transition to predominantly online gambling presents additional complexity in preventing and minimizing gambling harm for the public and individuals. To address the gaps identified above, and consistent with the comprehensive public health approach we have described, three broad areas of immediate action are identified to ameliorate the effects of gambling advertising and the impacts of the gambling industry in Ghana more broadly.

Firstly, the Commission’s advertising guidelines do not offer sufficient safeguards to protect the public, including young people and other vulnerable groups, from gambling harms. In the absence of countervailing action, this has led to aggressive marketing approaches and harmful practices by gambling operators as the competition for market share and profits intensifies. The ramifications have been the upward trend in gambling participation, especially among young males [7, 17, 18, 32] and other vulnerable groups. Addressing the ubiquity of gambling advertising requires strengthening the Commission to supervise the gambling industry proactively. Strategies could include adequately resourcing the Commission using full cost recovery mechanisms that would allow it to increase its fees and licenses proportional to the size of the gambling market [33]. Adequate resourcing would enable the Commission to effectively monitor and respond to the emergence of new technologies and practices, particularly those that pose a greater risk to young people.

Secondly, existing advertising guidelines point to the Commission’s objective to minimize young people's exposure to gambling advertising with clauses that ban advertisements during prime time and prohibit operators from using children’s songs or characters in advertisements [27]. While these are positive aspirations, the guidelines' apparent lack of enforcement is concerning. For example, gambling billboards can be found within 200 m of schools in a direct breach of the guidelines. Similarly, gambling advertisements are screened online during games on social media platforms, through direct messages via texts and emails. They are mentioned by sports commentators and presenters in live broadcasts, further contributing to the normalization of gambling for children and young people. There is the opportunity to reduce the exposure and appeal of gambling advertisements through restrictions on traditional, online, and digital gambling advertising. There is an urgent call to action for comprehensive advertising guidelines that set out clear prohibitions and expectations of advertising content and placement and clear plans for enforcement and monitoring.

Thirdly, a revised gambling advertising framework must prioritize upstream activities such as restricting all forms of advertising over the prevailing individual behavior approaches. We argue that a comprehensive public health approach [10] should underpin the new guidelines. The explicit aim should be to prevent gambling behavior, protect people from unsolicited and persistent gambling advertisements, minimize harm from gambling products, and ensure gambling advertising policies are independent of industry influence. Achieving this will require the Commission to shift its emphasis from promoting and protecting powerful vested interests that profit financially from gambling to becoming a proactive arbiter with a mandate to protect the public from harmful industry practices that drive gambling behavior. To support this aim, all operators should be obligated to age-verify all targeted online messages. Additional processes to apply appropriate age-gating technologies should be implemented to prevent underage young people from registering on gambling websites, apps, and social media accounts that promote gambling products. Consistent with findings from recent research on managing content relating to other harmful commodities, regulating online, digital and social media platforms should be prioritized to ensure greater accountability, transparency, content moderation, compliance and protection for young people and other vulnerable individuals [34]. Moreover, there should be strict adherence to the ban against celebrity endorsements. A contemporary gambling advertising guideline must explicitly ban social media influencers, content creators, and popular people on other online platforms from directly advertising (paid or commercial advertising) or organic (third-party) advertising of gambling products. Such action will assist to minimize exposure to gambling advertising and the risk of gambling uptake among underage and other vulnerable people [32]. Urgent public health advocacy is required to achieve new regulations that explicitly prohibit all forms of gambling advertising to protect children, young people, and other vulnerable groups- like tertiary students with visual impairments actively engaging in sport betting activities [35].

## Conclusions

Gambling advertising in Ghana is pervasive and anticipated to worsen as the industry focuses on new markets to grow and sustain profits. Doing nothing, as has been the case in the last decade, has reinforced the erroneous perception that gambling is not harmful. Inaction or weak regulation reinforces the notion that the government condones the harmful practices of the gambling industry and implicitly drives gambling behavior by protecting the interests of the industry over the health of the public. The consequent laissez-faire or letting people do as they choose approach to gambling advertising regulation has contributed to an environment where advertising is so widespread that there is significant exposure to young and other vulnerable people. As has been called for in other jurisdictions, a comprehensive public health approach is essential in responding to the gambling industry as a vector for disease. A focus on preventing gambling harm requires protecting the public from gambling marketing exposure and ensuring policies to regulate gambling are free from industry influence alongside efforts to minimize harm. In the Ghanaian context, useful next steps include instigating a review of the Gaming Act and the Commission that supervises the gambling industry to ensure it can supervise the gambling industry proactively. Importantly, a strengthened regulatory framework is imperative to restrict current advertising across all forms of media.
